# Reconstruction of facial defects using a pre-expanded scalp flap: A description of the method used and outcomes of 43 patients

**DOI:** 10.3389/fsurg.2022.962737

**Published:** 2022-08-08

**Authors:** Jianke Ding, Feifei Chu, Yinke Tang, Shiqiang Liu, Xianhui Zeng, Qing Yang, Xianjie Ma

**Affiliations:** ^1^ Department of Plastic and Reconstructive Surgery, Xijing Hospital, Fourth Military Medical University, Xi’an, China; ^2^ Department of Burn and Plastic Surgery, PLA No. 983 Hospital, Tianjin, China

**Keywords:** tissue expansion, postburn scar, facial defect, scalp flap, facial reconstruction

## Abstract

**Background:**

A technique for reconstructing facial units with matching colour, similar texture and sufficient contour is ideal for patients with various facial defects. The current report aimed to present the experience of the authors in facial reconstruction using pre-expanded scalp flaps combined with laser hair removal.

**Methods:**

From January 2014 to August 2021, 43 patients with different facial defects, such as post-burn scar and congenital nevus, were treated using this surgical technique that involved tissue expansion, scalp flap transfer and laser hair removal. Facial defects were artificially classified into three regions (forehead, *n* = 19; cheek, *n* = 15; and lips and chin, *n* = 9). Pedicle delaying and division were performed in patients who underwent reconstruction with pedicled flaps.

**Results:**

Of the included patients, one presented with haematoma, one with infection and three had distal necrosis after expanded scalp flap transfer. The donor site was primarily closed in all patients. Further, all patients were successfully treated without major complications. The texture, colour and contour of the scalp flap after laser hair removal matched well with the surrounding skin tissues at 2–30-month follow-up.

**Conclusion:**

Reconstruction using pre-expanded scalp flaps combined with laser hair removal is an effective and reliable option for facial reconstruction with excellent colour and texture match.

## Introduction

Facial defects, such as post-burn scars and congenital melanocytic nevus, affect patients both physically and mentally (1). However, as the reconstruction of such defects has strict requirements for facial appearance and function after surgery, it remains a challenge for plastic surgeons (2). Although skin grafting is a simple method, skin contracture and pigmentation or loss of skin grafts has a poor treatment outcome. Local flaps, such as facial rotation advancement flaps, require long back cut incisions. In contrast, free flaps are complicated and are associated with several risks, and transferred flaps require liposculpture due to their bulkiness (3).

The ultimate goals of facial reconstruction have virtually remained unchanged, and these include complete removal of all lesions and their replacement with tissues possessing qualities similar to those of the remaining face and surrounding regions. Despite advancements in facial reconstruction, achieving satisfactory functional and aesthetic outcomes remains a surgical challenge (4). Pre-expansion of flaps can provide large, thin and pliable skin tissues and enables the reconstruction of the head and neck contours (5). Previous studies have reported the use of expanded facial, deltopectoral flaps and expanded free flaps. However, the application of these flaps is limited if a donor site is not available (6, 7).

A previous study reported that the quality, colour and texture of the scalp flap used after hair removal were similar to those of facial skin tissues (8). The pre-expansion of scalp flaps not only provides sufficient skin tissues for facial reconstruction but also facilitates the primary closure of donor sites. Therefore, the current report aimed to assess whether all facial units, including the forehead, cheek and perioral region (lips and chin), can be aesthetically managed *via* reconstruction with expanded scalp flaps combined with laser hair removal. Thus, we retrospectively summarized the application of pre-expanded scalp flaps in facial reconstruction at our centre from 2014 to 2021.

## Materials and methods

### Patients

From January 2014 to August 2021, 43 patients [24 men and 19 women, aged 3–42 years (average: 19 years)] who presented with facial defects underwent reconstruction using expanded scalp flaps combined with laser hair removal. Of these 43 patients, 30 had post-burn scar, 12 had congenital melanocytic nevus and 1 had verrucous nevus (Table 1). For better guidance, the facial defects were artificially classified into three regions (the forehead, *n* = 19; the cheek, *n* = 15; and the lips and chin, *n* = 9). The size of the regions with facial defects ranged from 3 × 5 cm^2^ to 12 × 25 cm^2^. The patients underwent tissue expansion, scalp flap transfer, pedicle division and laser hair removal. This non-experimental, retrospective report was approved by the institutional review board of the institution, and informed consent was obtained from each patient.

**Table 1:** The cause of the patients in our study.

Cause	Postburn scar	Congenital melanocytic nevus	Verrucous nevus
Patients, *n*	30	12	1

### Preoperative preparation

The shape and size of the expanders should be estimated according to the size and location of the facial defect. In general, a 100- to 1,000-ml rectangular or cylindrical tissue expander was selected. According to facial defect locations, three types of expanded scalp flaps were adopted. To treat the lesions on the forehead, the expanders were implanted under the scalp near the defect; to treat those on the cheek or upper lip, they were implanted under the temporal scalp; and to treat lesions on the lower lip and chin, they were implanted under the top of the scalp. An ultrasound Doppler probe was used to explore the location and course of the superficial temporal vessels, which were marked to help identify the flap pedicle.

### Expander implantation

To insert the expander, a 3–8-cm horizontal incision 2 cm within the hairline were made. The expanders were implanted in the pocket between the epicranial aponeurosis and periosteum of the cranium, and the pocket was 1 cm wider than the margin of the selected expander. The wound was vacuum-drained for 2–3 days until the fluid became clear and daily volume was negligible. The expander was inflated on postoperative day 3 or 5, twice a week, by injecting normal saline until adequate volume had been reached. Generally, it took about 2–3 months before flap transfer.

### Expanded scalp flap transfer

**Figure 1:**
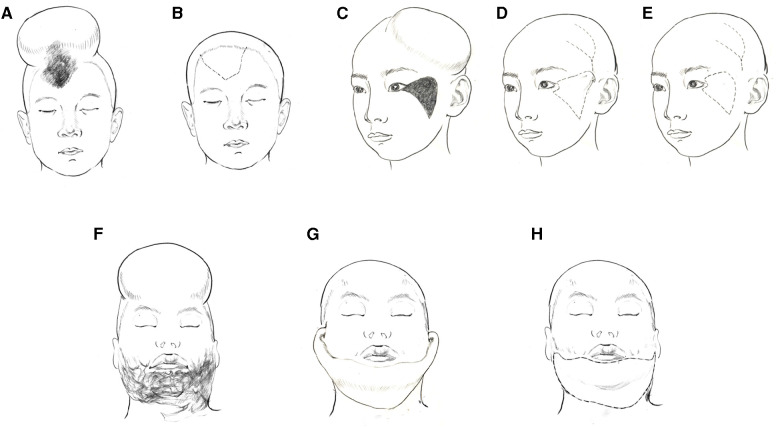
Schematic diagram of the operation method according to the location of the lesions. (**A,B**), The expanded scalp flaps were transferred to the defect *via* flap advancement to treat forehead defects. (**C–E**), the expanded scalp flaps were raised and transferred to reconstruct the cheek or upper lips pedicled by the unilateral superficial temporal artery. (**F–H**), the expanded scalp flaps were raised and transferred pedicled by both the superficial temporal vessels to treat lesions on the lower lip and chin.

Facial defects were first excised and completely released. Each scalp flap was designed according to the location and shape of the facial defect (Table 2). The expanded scalp flaps were transferred to the defect *via* flap advancement to treat forehead defects (Figures 1A,B). Based on the unilateral superficial temporal artery, these flaps were raised and transferred to reconstruct the cheek or upper lips (Figures 1C–E). Both the superficial temporal vessels should be included along the longitudinal axis of the designed flap to treat lesions on the lower lip and chin (Figures 1F–H). The pedicled expanded scalp flaps were raised, thereby retaining the superficial temporal vessels and scalp tissues as their pedicle. The proximal pedicle tissues were either tubularised or covered *via* split skin grafting and separated 3 weeks after surgery.

**Table 2:** The location of the defects and the surgical method of the patients.

Defect location	Forehead	Cheek and upper lip	Chin and lower lip
Patients, *n*	30	12	9
Surgical methods	Local flap transfer	Single pedicle flap	Bipedicle flap

In general, the actual flap size should be 10%–15% larger than that of the facial defect because the expanded scalp will exhibit instant contraction immediately after the removal of the expander. The wound in the donor site was primarily closed, and a drainage tube was placed underneath the flap. A pressure dressing was applied over the flap region to prevent tissue swelling and heamatoma.

### Laser hair removal

Using the transferred scalp flap, the hairline, eyebrow and beard of the patients could be reconstructed. Therefore, personalised customisation is needed when opting for laser hair removal, which is typically performed within 2 weeks after scalp flap transfer. An 800 nm diode laser (LightSheer Duet, Lumenis Ltd., Santa Clara, CA, the USA) was used with a fluence of 16–24 J/cm^2^ and pulse duration of 30 ms. The spot size was 9 mm × 9 mm. The treatment interval was 25–30 days. The initial fluence was preferably low, and the subsequent fluence was adjusted according to hair thickness. Each patient underwent 2–6 sessions of treatment based on their individual conditions. Ice packs were placed over the laser-treated regions for 30–60 min to relieve laser-induced local pain and swelling.

## Results

During the tissue expansion period, one patient presented with haematoma and was cured *via* the removal of haematoma and the re-implantation of tissue expander. Another patient developed infection and received early flap transfer without subsequent complications. None of them presented with expander exposure. Distal necrosis was observed in three patients after expanded scalp flap transfer. One patient was cured using a full-thickness skin graft, and two were treated *via* daily dressing changes (Table 3). The size of the region where the scalp flap was transferred ranged from 5 × 5 cm^2^ to 13 × 30 cm^2^. After 2–6 sessions of laser hair removal treatment, the texture, colour and contour of the transferred scalp flap matched well with the surrounding skin tissues. The donor sites were primarily closed in all patients, with minimal morbidity at the donor site. The aesthetic clinical outcome was achieved at the 2–30-month follow-up.

**Table 3:** Complications.

Type of complications	Patients, *n* (%)
expander exposure	–
Hematoma	1, (2.3)
Infection	1, (2.3)
Distal necrosis of scalp flap	3, (7.0)

## Case reports

### Case 1

**Figure 2:**
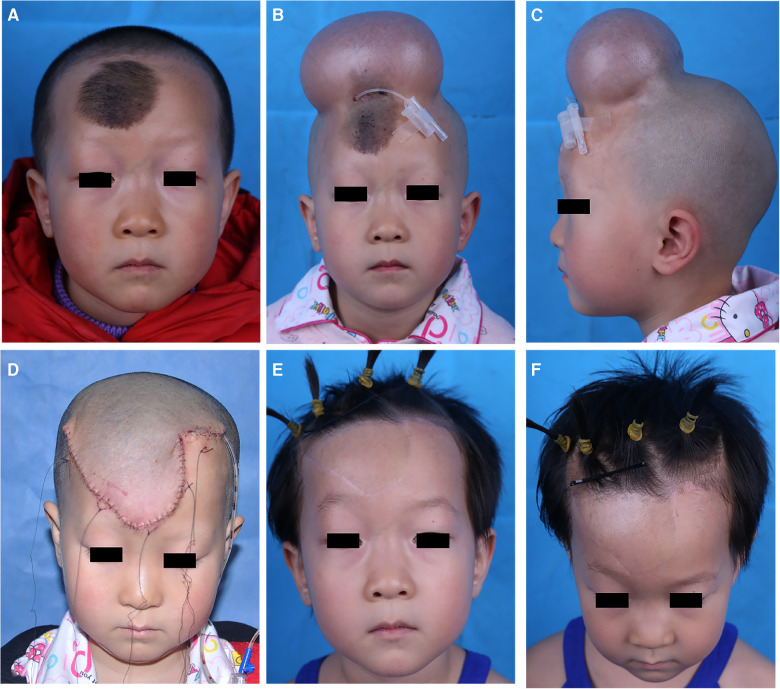
Case 1 (**A**), a 4-year-old girl with congenital nevus (5 × 4 cm^2^) on forehead. (**B,C**), a 300 ml cylindrical expander was implanted under the scalp near the lesion, the final expansion volume was 415 ml. (**D**), the expander was removed and expanded scalp flap was transferred to cover the facial defect. (**E,F**), 5-months postoperative view after 3 sessions of laser hair removal.

A 4-year-old girl presented with a congenital black, hairy patch (5 × 4 cm^2^) on the forehead (Figure 2A). To reconstruct the defect after excising the patch, a 300-mL cylindrical expander was placed under the scalp near the lesion. After 3 months, the final expansion volume was 415 ml (Figures 2B,C). Following the removal of the expander, a scalp flap (6 × 5 cm^2^) matching the defect in size and shape was raised for transfer to the forehead by direct advancement. (Figure 2D). The patient underwent postoperative laser hair removal 3 times, and the texture and colour of the transferred scalp flap were similar to those of the adjacent skin at the 5-month follow-up (Figures 2E,F).

### Case 2

**Figure 3:**
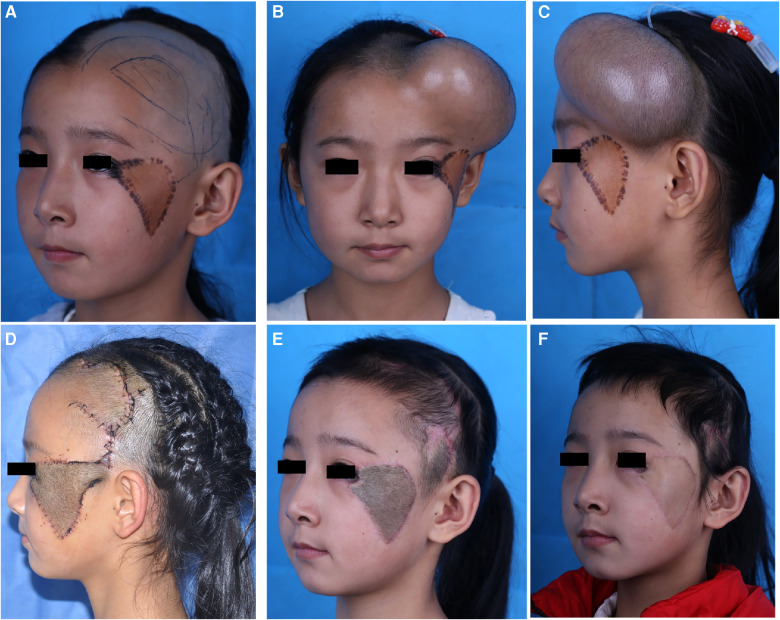
Case 2 (**A**), a 10-year-old girl with recurrent nevus and hyperpigmentation on left cheek. (**B,C**), a 200 ml cylindrical expander was implanted under temporal scalp, the final expansion volume was 300 ml in 2 months. (**D**), the scalp flap based on superficial temporal artery was raised and transferred to reconstruct facial defect after the expander was removed. (**E**), Postoperative view after pedicle delay and division. (**F**), 10-month follow up after 3 sessions of laser hair removal.

A 10-year-old girl presented with recurrent nevus and hyperpigmentation on the left cheek after skin grafting for facial congenital nevus reconstruction (Figure 3A). To treat the lesion on the cheek, a 200-mL cylindrical expander was implanted under the temporal scalp. The expander was inflated with saline solution, reaching a volume of 300 ml in 2 months (Figures 3B,C). Then, based on the unilateral superficial temporal artery, the planned scalp flap was raised and transferred to reconstruct the wound after lesion excision. The wound at the donor site was directly closed intraoperatively, and the proximal pedicle tissue was tubularised (Figure 3D). Pedicle delay and division surgeries were performed after 3 weeks (Figure 3E). Laser hair removal was started at 3 weeks after the division surgery, and excellent outcomes were achieved at 8 months after the surgery (Figure 3F).

### Case 3

**Figure 4:**
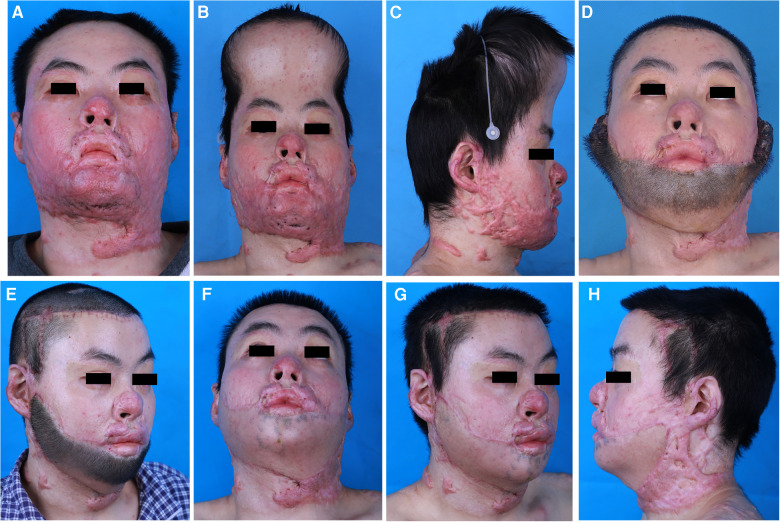
Case 3 (**A**), a 20-year-old man with severe post-burn scars in his lips and chin. (**B,C**), an 800 ml expander was implanted under the scalp. After two months, the final expansion volume was 1090 ml. (**D**), the expanded scalp flap based on bilateral superficial temporal vessels was transferred to repair lower lip and chin. (**E**), postoperative view of 10 days after pedicle division. (**F–H**), the results at 5 months post-surgery after 2 sessions of laser hair removal treatment were aesthetically satisfactory.

A 20-year-old man presented with severe post-burn scars in his lips and chin (Figure 4A). To treat the lesions on the lower lip and chin, an 800 ml expander was implanted under the scalp. After skin expansion for 2 months *via* regular injection of saline solution, the final expansion volume was 1,090 ml (Figures 4B,C). The bilateral superficial temporal vessels were marked using Doppler ultrasonography, and a safe width for the pedicle was designed for either side preoperatively. After the lesions on the lower lip and chin were excised, the expanded scalp flap (28 × 10 cm^2^) was elevated and transferred to the recipient site (Figure 4D). The pedicles containing skin tissues were carefully harvested on both sides, and dressings were changed daily before performing divisions. The transferred scalp flap was used to reconstruct the lower lip and chin. Excellent outcomes were achieved at 5 months postoperatively (Figures 4F–H).

## Discussion

In facial reconstruction, matching the colour and texture are the primary considerations. Facial defect reconstruction *via* skin grafting often results in hyperpigmentation and contracture, particularly in Asian patients. Facial defect repair using local skin flaps could increase linear scars along the adjuvant incisions. Further, expanded distant deltopectoral flaps are used in facial reconstruction. However, scars on the donor chest are difficult to accept particularly among young women (5). Therefore, exploring a reliable and practicable method for facial repair and reconstruction is clinically important.

Traditionally, the pre-expanded scalp flap is a dependable option for treating scarring alopecia due to its reliable blood supply and inconsequential scarring at the donor sites (9). The combination of scalp flap transfer and laser hair removal has recently been used for forehead repair and nasal reconstruction. The colour and texture of the scalp after hair removal were similar to those of facial skin tissues (8, 10, 11). Besides few relevant case reports, some studies in the literature systematically revealed the application of an expanded scalp flap in facial reconstruction in a significant number of cases. In this report, 43 patients underwent facial defect repair using expanded scalp flaps combined with laser hair removal, demonstrating the viability of expanded scalp flap in the reconstruction of almost all facial units, such as the forehead, cheek and perioral region (lips and chin).

Tissue expansion is a common surgical procedure performed by plastic surgeons to harvest extra skin for resurfacing defects. According to a previous report, scalp expansion showed a relatively low incidence of relevant complications during the expansion period due to the texture of the skull (12). In addition, blood supply to the scalp is abundant, and either the unilateral or bilateral superficial temporal vessels could be used as a pedicle for further scalp flap transfer (13). The thickness of scalp flaps after expansion was suitable for resurfacing facial defects without the need for subsequent flap debulking. However, tissue expansion has few limitations, such as multi-staged procedure, prolonged hospitalisation time and high costs.

According to a previous study, the superficial temporal artery has thin venae comitantes that give off several branches towards the skin and underlying soft tissues (14). In our patients, we retained the skin tissues of the pedicles to ensure venous protection and drainage to the anatomical tissues. Then, delayed treatment of the transferred flap was performed by transecting the skin tissues of the pedicle after 3 weeks. Notably, our method ensured a reliable blood supply to the pedicled scalp flaps. Of the 43 patients in our report, none presented with extensive flap necrosis, but three had distal necrosis of the flap.

Laser techniques are rapidly upgrading. Laser devices, such as diode laser (800 nm), long-pulsed alexandrite laser (755 nm) and Nd:YAG laser (1,064 nm), are widely used in laser hair removal (15), which utilises red or near-infrared light to target melanin in hair follicles and generate thermal energy to damage these follicles and consequently impair hair growth. In this report, we performed a diode laser treatment in patients within 2 weeks after the surgery as microcirculation was not fully established in the hair follicles. Satisfactory outcomes were achieved using laser hair removal in 2–6 treatment sessions.

## Conclusions

Reconstruction using pre-expanded scalp flaps combined with laser hair removal is an effective and reliable option for repairing facial defects. Although this approach involves a multi-staged procedure, it can be used for reconstructing facial defects with skin tissues that are from a single donor site and possess qualities matching those of the remaining face. Therefore, the pre-expanded scalp flap can be widely used in the repair of all facial defects.

## Data Availability

The raw data supporting the conclusions of this article will be made available by the authors, without undue reservation.
